# Getting the LV lead to the right spot

**DOI:** 10.1007/s12471-015-0774-6

**Published:** 2015-12-08

**Authors:** K.P. Jackson, T. Steen

**Affiliations:** 1 Department of Medicine, Duke University Medical Center, Durham, NC USA; 2 Pacemaker & ICD Centre, Oslo University Hospital Ullevaal, Oslo, Norway

In an acute study of epicardial (transvenous, via the coronary sinus, CS) and endocardial left ventricular (LV) pacing in clinical non-responders to CRT, Van Gelder et al. found that endocardial pacing at a late activated site could improve LV function, measured by LV dP/dT [1]. There was, however, no difference between endocardial and epicardial pacing at the same anatomic location. Thus, the beneficial effect of endocardial pacing in this study appeared to be the ability to pace the latest activated part of the left ventricle, rather than the endocardium *per se*. So, while we are waiting for endocardial LV pacing to possibly mature as a clinical tool, we should strive to improve our ability to get transvenous, epicardial leads to the best spot.

The importance of LV lead position to long-term outcomes has been demonstrated in several trials [2, 3]. Improved survival is seen when the LV lead is placed in the basal or mid-portion of the lateral or posterolateral region, or the lead is targeted to the latest mechanically activated region [4]. CS anatomy may present multiple challenges that make targeted LV lead placement difficult. In the present study, of the 11 patients showing an acute haemodynamic response to LV pacing at an alternate position, 9 had CS leads placed in sub-optimal positions (apical or anterior). Placement of LV leads in narrow or tortuous branches of the CS may require specialised techniques. In a study comparing the standard (‘over the wire’) LV lead implant technique to an interventional technique using a telescoping system of catheters and contrast injection, the interventional technique resulted in higher procedural success, significantly shorter fluoroscopy times and a higher percentage of LV leads located in optimal regions of the LV wall [5]. The improvements in procedural outcomes were likely due to (1) rapid identification of the CS ostium with contrast, (2) rapid access to the target branch with angled, directional catheters, and (3) improved stability for lead advancement from direct catheter support in the target branch.

The interventional technique for LV lead implantation is shown in Fig 1. The CS ostium is localised using contrast injected through a 7-Fr braided CS access catheter (panel A). A 9-Fr inner-diameter sheath is advanced over this catheter directly into the CS. Once the target branch is identified, cannulation is performed with a two-component, telescoping lead delivery system consisting of a target vein selector connected to the contrast injection system and a delivery guide shaped to fit into the target vein and deliver the LV lead (panel B). Advancement of the LV lead using this system is through the delivery guide catheter placed directly into the target branch, and is guided but not dependent upon wire support (panel C and D).

**Fig. 1:**
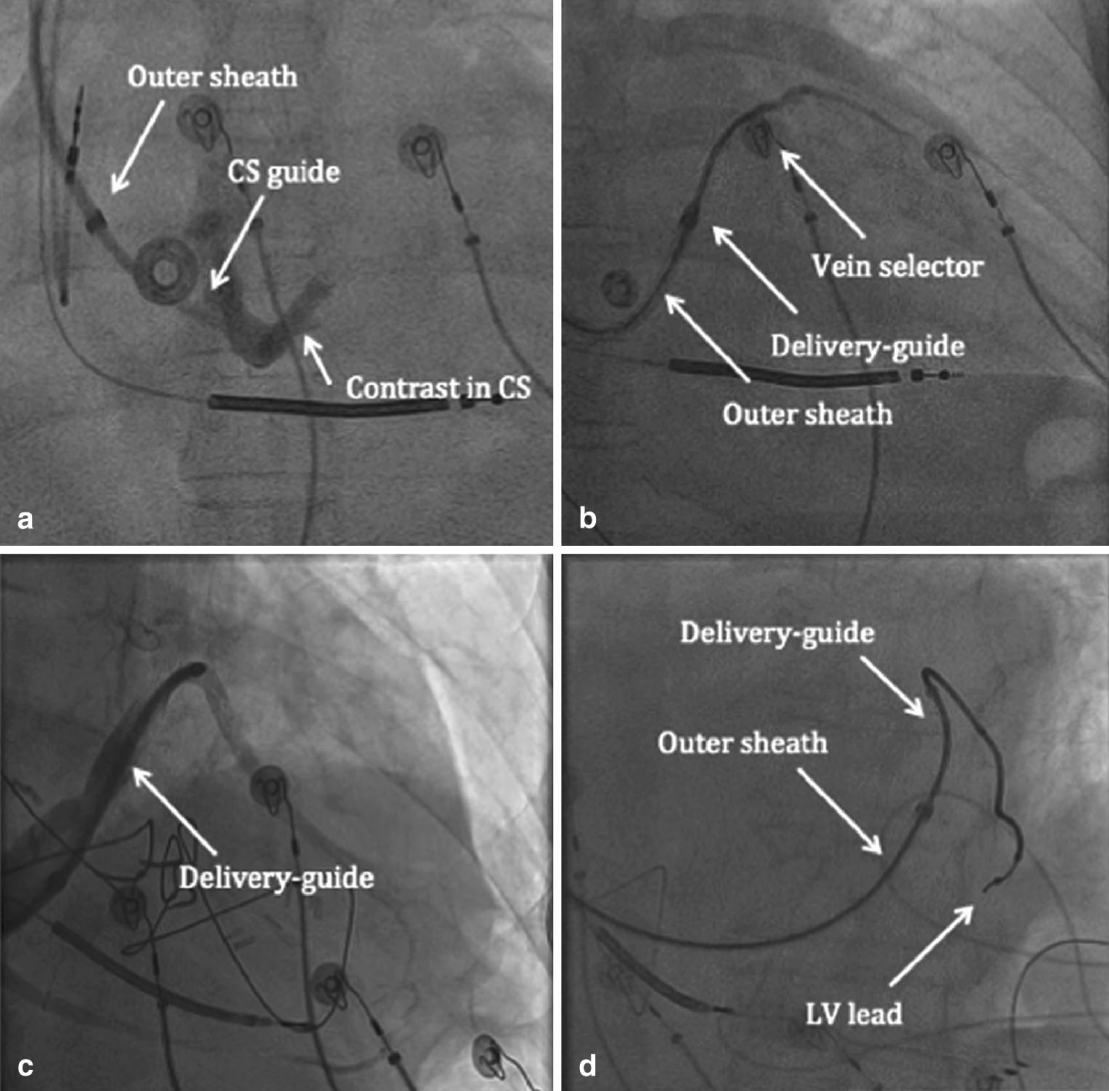
Interventional implant technique for LV lead placement is shown. See text for details

If cannulation of the target vein does not give enough support to advance the lead in a tortuous or narrow vein, a snare can be used: A hydrophilic 0.014” wire is advanced from the target vein through collaterals back into the CS. A 10 mm 4-Fr gooseneck snare is advanced through the CS guide, and used to hold the distal end of the wire. The lead is then advanced over the wire, which now cannot possibly prolapse. A 9-Fr CS guide must be used to take both the 4-Fr snare and a 5-Fr lead. Alternatively, the wire can be snared with a 40 mm basket snare in the right atrium, introduced with a separate venous access via the axillary/subclavian vein. But getting the wire into the snare is much easier in the CS. If a stenotic target vein prevents lead advancement, one can perform venoplasty with a PCI balloon. Firm support, either from the deep-seated, soft, 7-Fr target vein catheter or from a snared wire, is useful. If the target vein is tortuous or has a difficult take off, one or more additional, stiffer 0.014” wires (‘buddy wires’) can provide the necessary support. The target vein catheter must be 7-Fr to take the buddy wires alongside the lead. Entering a target vein close to the CS ostium can be difficult. Using a 9-Fr guide, a stiff, soft-tipped, 0.035” wire up into the CS can be used as a ‘support wire’, to prevent the CS guide from falling out. The target vein can then be cannulated by a 5-Fr ‘vein selector’ catheter, which is removed after one or more 0.014” wires are introduced. There is room for the 0.035” support wire and the lead if the CS guide is 9-Fr, so the support wire can be removed after the lead is well in place and the CS guide has been taken out. If the subclavian or brachiocephalic vein is stenotic, which is often the case with ‘upgrades’, venoplasty with a 6 × 40 mm balloon can give the necessary access for the CS guide. This is easy to learn and safe, provided simple safety measures are taken.

Endocardial LV lead placement via transseptal puncture is a recently described, alternate technique when the lead cannot be placed in an adequate branch via the CS and surgical lead placement is not possible or desired. In the current study, temporary LV lead placement and haemodynamic measurements were performed via standard transseptal access from the femoral vein (or radial artery in the case of one patient). However, when the decision to place a permanent transseptal LV lead was reached (in 5 patients), careful procedural planning and consideration of deleterious effects of this approach were considered. While technically feasible, placement of an endocardial LV lead from the subclavian vein requires specialised tools such as snares to direct a sheath across a transseptal puncture from a superior approach. In addition, the pro-thrombotic effects of endocardial LV lead placement are unknown. In the absence of better tools and better data on transseptal lead placement, an attempt at an alternate LV lead location via the CS would seem warranted.

In conclusion, learning to use the interventional techniques and the custom tools discussed above will improve the ability to get the epicardial lead to the best spot. In that way, one can increase response rate and reduce the number of cases where surgical LV lead placement or endocardial LV pacing is necessary.

## Disclosures

K.P. Jackson: Consulting fees/honorarium from Medtronic and Merit Medical Systems.

T. Steen: Merit Medical: Speakers fee for lecture on interventional CRT in Milan, 20 October 2015. Visited Dr. Seth Worley in Lancaster in 2012 to learn the techniques. The expenses were paid by myself and by Oslo University Hospital. Seth Worley was an invited guest operator and lecturer at Oslo University Hospital twice, in 2013 and 2014. Merit Medical and Octopus Medical paid his travel expenses.
